# Ligand-Based Virtual Screening Using Bayesian Inference Network and Reweighted Fragments

**DOI:** 10.1100/2012/410914

**Published:** 2012-05-01

**Authors:** Ali Ahmed, Ammar Abdo, Naomie Salim

**Affiliations:** ^1^Faculty of Computer Science and Information Systems, Universiti Tecknologi Malaysia, 81310 Skudai, Malaysia; ^2^Faculty of Engineering, Karary University, Khartoum 12304, Sudan; ^3^Department of Computer Science, Hodeidah University, Hodeidah, Yemen

## Abstract

Many of the similarity-based virtual screening approaches assume that molecular fragments that are not related to the biological activity carry the same weight as the important ones. This was the reason that led to the use of Bayesian networks as an alternative to existing tools for similarity-based virtual screening. In our recent work, the retrieval performance of the Bayesian inference network (BIN) was observed to improve significantly when molecular fragments were reweighted using the relevance feedback information. In this paper, a set of active reference structures were used to reweight the fragments in the reference structure. In this approach, higher weights were assigned to those fragments that occur more frequently in the set of active reference structures while others were penalized. Simulated virtual screening experiments with MDL Drug Data Report datasets showed that the proposed approach significantly improved the retrieval effectiveness of ligand-based virtual screening, especially when the active molecules being sought had a high degree of structural heterogeneity.

## 1. Introduction

Virtual screening refers to the use of a computer-based method to process compounds from a library or database of compounds in order to identify and select ones that are likely to possess a desired biological activity, such as the ability to inhibit the action of a particular therapeutic target. The selection of molecules with a virtual screening algorithm should yield a higher proportion of active compounds, as assessed by experiment, relative to a random selection of the same number of molecules [1]. 

Over recent decades, drug discovery companies have used combinatorial chemistry approaches to create large and diverse libraries of structures; therefore large arrays of compounds are formed by combining sets of different types of reagents, called building blocks, in a systematic and repetitive way. These libraries can be used as a source of new potential drugs, since the compounds in the libraries can be randomly tested or screened to find good drug compounds. Increasing the capabilities of testing compounds using chemoinformatic technologies such as high-throughput screening (HTS) enables hundreds of thousands of these compounds to be tested in a short time. Computers can be used to aid this process in a number of ways; for example, in the creation of virtual combinatorial libraries which can be much larger than their real counterparts. There are two methods for screening those libraries, looking into active sites of interest and looking for similarities to a known active compound. Recently, searching chemical databases has been done using computers instead of experiment, and this is known as the virtual screening technique [2–9].

Chemical information systems offer three principal types of searching facility. Early systems provided two types of retrieval mechanisms: structure searching and substructure searching. These mechanisms were later complemented by another access mechanism: similarity searching. There are many studies in the literature associated with the measurement of molecular similarity [10–13]. However, the most common approaches are based on 2D fingerprints, with the similarity between a reference structure and a database structure computed using association coefficients such as the Tanimoto coefficient [1, 14].

Several methods have been used to further optimise the measures of similarity between molecules, including weighting, standardization, and data fusion [15–18].

The Bayesian inference network (BIN) was originally developed for text document retrieval systems [19]. Many studies in information retrieval (IR) have shown that the retrieval effectiveness of BIN can be improved by fragment reweighting. Fragments reweighting is one of the most useful query modification techniques in IR systems [20–22]. In our previous works, the retrieval performance of Bayesian inference network was observed to improve significantly when relevance feedback and turbo search screening were used [23].

In this paper, we enhanced the screening effectiveness of BIN using a weighting factor. In this approach, weighting factors are calculated for each fragment of the multireference input query based on the frequency of their occurrence in the set of references' input. This weighting factor is later used to calculate a new weight for each fragment of the reference structure.

## 2. Material and Methods

This study has compared the retrieval results obtained using three different similarity-based screening models. The first screening system was based on the tanimoto (TAN) coefficient, which has been used in ligand-based virtual screening for many years and is now considered a reference standard. The second model was based on a basic BIN [24] using the Okapi (OKA) weight, which was found to perform the best in their experiments and which we shall refer to as the conventional BIN model. The third model, our proposed model, is a BIN based on reweighted fragments, which we shall refer to as the BINRF model. In what follows, we give a brief description of each of these three models.

### 2.1. Tanimoto-Based Similarity Model

This model used the continuous form of the tanimoto coefficient, which is applicable to nonbinary data of fingerprint. *S*_*K*,*L*_ is the similarity between objects or molecules *K* and *L*, which, using tanimoto, is given by (1):

(1) $${\text{S}}_{kL}=\frac{\sum_{j=1}^{M}w_{jk}w_{jl}}{\sum_{j=1}^{M}{\left(w_{jk}\right)}^{2}+\sum_{j=1}^{M}{\left(w_{jl}\right)}^{2}-\sum_{j=1}^{M}\left(w_{k}w_{jl}\right)}.$$

For molecules described by continuous variables, the molecular space is defined by an *M* × *N* matrix, where entry *w*_*ji*_ is the value of the *j*th fragments (1 ≤ *j* ≤ *M*) in the *i*th molecule (1 ≤ *i* ≤ *N*). The origins of this coefficient can be found in a review paper by Ellis et al. [25].

### 2.2. Conventional BIN Model

**Figure 1:**
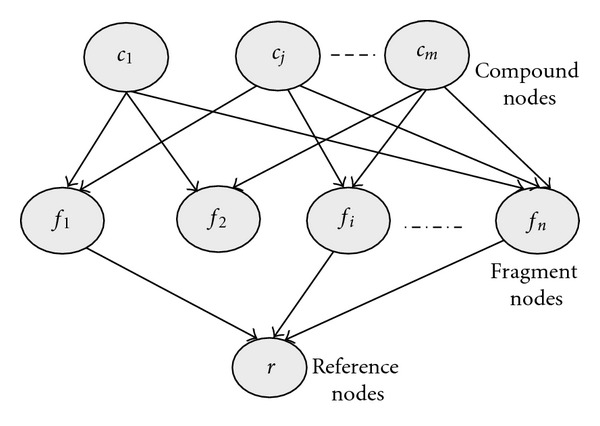
Bayesian inference network model.

The conventional BIN model, as shown in Figure 1, is used in molecular similarity searching. It consists of three types of nodes: compound nodes as roots, fragment nodes, and a reference structure node as leaf. The roots of the network are the nodes without parent nodes and the leaves are the nodes without child nodes. Each compound node represents an actual compound in the collection and has one or more fragment nodes as children. Each fragment node has one or more compound nodes as parents and one reference structure node as a child (or more where multiple references are used). Each network node is a binary value, taking one of the two values from the set {true, false}. The probability that the reference structure is satisfied given a particular compound is obtained by computing the probabilities associated with each fragment node connected to the reference structure node. This process is repeated for all the compounds in the database.

The resulting probability scores are used to rank the database in response to a bioactive reference structure in the order of decreasing probability of similar bioactivity to the reference structure.

To estimate the probability associating each compound to the reference structure, the probability for the fragment and reference nodes must be computed. One particular belief function, called OKA, has been found to have the most effective recall [24]. This function was used to compute the probabilities for the fragment nodes and is given by (2):

(2) $$\begin{array}{cc} \text{be}{\text{l}}_{\text{OKA}}\left(f_{i}\right) & =\alpha +\left(1-\alpha \right)\times \frac{ff_{ij}}{ff_{ij}+0.5+1.5\times \left|c_{j}\right|/\left|{\text{c}}_{\text{avg}}\right|}\\ & \;\times \frac{\log\left[\left(m+0.5\right)/cf_{i}\right]}{\log\left(m+1.0\right)}, \end{array}$$

where *α* = Constant; and experiments using the BIN show that the best value is 0.4 [26, 27], *ff*_*ij*_ = frequency of the *i*th fragment within the *j*th compound reference structure, *cf*_*i*_ = number of compounds containing the *i*th fragment, |*c*_*j*_|= the size (in terms of number of fragments) of the *j*th compound, |*C*_avg_| = the average size of all the compounds in the database, and *m* = the total number of compounds. 

To produce a ranking of the compounds in the collection with respect to a given reference structure, a belief function from In Query, the SUM operator, was used. If *p*1, *p*2,…, *pn* represent the belief in the fragment nodes (parent nodes of *r*), then the belief at *r* is given by (3):

(3) $${\text{bel}}_{\text{sum}}\left(r\right)=\frac{\sum_{i=1}^{n}p_{i}}{n},$$

where *n* = the number of the unique fragments assigned to reference structure *r*.

### 2.3. BINRF Model

The difference between the two models (BIN and BINRF) arises from the differences in the type of belief function used to produce the ranking of the compounds in the collection. In the conventional BIN model, the probability of the reference node is computed by summing the probabilities in the fragment nodes connected to the reference node. The fragment nodes participating in the final probability are scored equally (meaning that no extra weight given to any fragment node). This calculation is conducted using the SUM operator, as described above.

In the BINRF model, the reweighting factor is used to assign a new weight to the fragment. In order to produce this factor, it is necessary to start by analysing the occurrence of each fragment in the set of input references. The reweighing factor *rwf*_*i*_  is calculated using (4):

(4) $${rwf}_{i}=\frac{F_{f_{i}}}{\max F},$$

where *F*_*f*_*i*__ is the frequency of *i*th fragment in the set of references' input and max⁡*F* is the maximum fragment frequency in the set of references' input.

New weights are then assigned to the fragments based on this factor, the new weight, *nw*_*i*_, of the *i*th fragment, is given by (5):

(5) $${nw}_{i}=w_{i}+rwf_{i},$$

where *w*_*i*_ is the original frequency of the *i*th fragment in the reference input.

Consequently, the use of (4) and (5) to assign the new weights shows that higher weights will be assigned to those that occur more frequently in the set of references' input structures.

### 2.4. Experimental Design

The searches were carried out on the MDL Drug Data Report (MDDR) database. The 102,516 molecules in the MDDR database were converted to Pipeline Pilot ECFC_4 fingerprints and folded to give 1024-element fingerprints [28].

For the screening experiments, three data sets (DS1–DS3) [29] were chosen from the MDDR database. Dataset DS1 contains 11 MDDR activity classes, with some of the classes involving actives that are structurally homogeneous and others involving actives that are structurally heterogeneous (structurally diverse). The DS2 dataset contains 10 homogeneous MDDR activity classes and the DS3 dataset contains 10 heterogeneous MDDR activity classes. Full details of these datasets are given in Tables 1–3. Each row in the tables contains an activity class, the number of molecules belonging to the class, and the class's diversity, which was computed as the mean pair-wise Tanimoto similarity calculated across all pairs of molecules in the class using ECFP6. The pair-wise similarity calculations for all datasets were performed using Pipeline Pilot software [28]. 

For each dataset (DS1–DS3), the screening experiments were conducted with 10 reference structures selected randomly from each activity class and the similarity measure used to obtain an activity score for all of its compounds. These activity scores were then sorted in descending order with the recall of the active compounds, meaning the percentage of the desired activity class compounds that are retrieved in the top 1% and 5% of the resultant sorted activity scores, providing a measure of the performance of our similarity method.

## 3. Results and Discussion

Our goal was to identify different retrieval effectiveness of using different search approaches. In this study, we tested the TAN, BIN, and BINRF models against the MDDR database using three different datasets (DS1–DS3). The results of the searches of DS1–DS3 are presented in Tables 4-6, respectively, using cutoffs at both 1% and 5%.

In these tables, the first column from the left contains the results for the TAN, the second column contains the corresponding results when BIN is used, and the last column of each table contains the corresponding results when BINRF is used.

Each row in the tables lists the recall for the top 1% and 5% of a sorted ranking when averaged over the ten searches for each activity class; and the penultimate row in each table corresponds to the mean value for that similarity method when averaged over all of the activity classes for a dataset. The similarity method with the best recall rate in each row is strongly (∗∗), and the recall value is boldfaced; any similarity method with an average recall within 1% and 5% of the value for the best similarity method is shown lightly (∗). The bottom row in a table corresponds to the total number of (∗ and ∗∗) cells for each similarity method across the full set of activity classes. 

Visual inspection of the recall values in Tables 4–6 enables comparisons to be made between the effectiveness of the various search models. However, a more quantitative approach is possible using the Kendall *W* test of concordance [30].

This test shows whether a set of judges make comparable judgments about the ranking of a set of objects; here, the activity classes were considered the judges and the recall rates of the various search models the objects. The outputs of this test are the value of the Kendall coefficient and the associated significance level, which indicates whether this value of the coefficient could have occurred by chance. If the value is significant (for which we used cutoff values of both 0.01 and 0.05), then it is possible to give an overall ranking of the objects that have been ranked. The results of the Kendall analyses (for DS1–DS3) are reported in Table 7 and describe the top 1% and top 5% rankings for the various weighting functions. In Table 7, the columns show the dataset type, the recall percentage, the value of the coefficient, the associated probability, and the ranking of the methods.

Some of the activity classes, such as low-diversity activity classes, may contribute disproportionally to the overall value of mean recall. Therefore, using the mean recall value as the evaluation criterion could be impartial in some methods but not in others. To avoid this bias, the effective performances of the different methods have been further investigated based on the total number of (∗ and ∗∗) cells for each method across the full set of activity classes, as shown in the bottom rows of Tables 4–6. These (∗ and ∗∗) cell results are also listed in Table 8 (the results shown in the bottom rows of Tables 4–6 form the lower part of the results in Table 8).

Inspection of the DS1 search in Table 4 shows that BINRF produced the highest mean values when compared to the BIN and TAN. In addition, according to the total number of (∗ and ∗∗) cells in Table 4, BINRF is the best performing search across the 11 activity classes in terms of mean recall. Table 7 shows that the value of the Kendall coefficient for DS1 top 1% and 5%, 0.752, is significant at the 0.01 and 0.05 levels of statistical significance. Given that the result is significant, we can conclude that the overall ranking of the different procedures are BINRF > BIN > TAN and BINRF > TAN > BIN for the DS1 top 1% and 5%, respectively.

 The good performance of the BINRF method is not restricted to DS1 since it also gives the best results for the top 1% and 5% for DS2 and DS3.

The DS3 searches are of particular interest since they involve the most heterogeneous activity classes in the three datasets used, and thus provide a tough test of the effectiveness of a screening method. Hert et al. [29] found that TSS (group fusion) was not preferred to the conventional similarity search for DS3 activity classes. However, when BINRF is used on this dataset, Tables 6 and 7 show that it gives the best performance of all the methods for this dataset at both cutoffs.

Visual inspection of the results in Tables 4–8 shows very clearly that reweighting reference fragments can significantly increase the effectiveness of the BIN method and the results are presented for the original search using TAN, BIN, and BINRF. A very surprising pattern of behaviour is observed in the DS3 results presented in Table 6 as the degree of enhancement in this more challenging screening task is remarkable.

In conclusion, we have introduced a new technique for utilising the effectiveness of retrieval when applying a BIN for ligand-based virtual screening. Simulated virtual screening experiments with MDDR datasets showed that the proposed techniques described here provide simple ways of enhancing the cost effectiveness of ligand-based virtual screening in chemical databases.

## 4. Conclusion

In this paper, we further investigated the impact of reweighting fragments on the Bayesian inference network performance for ligand-based virtual screening. Simulated virtual screening experiments with MDL Drug Data Report datasets showed that the proposed approach significantly improved the retrieval effectiveness of ligand-based virtual screening, especially when the active molecules being sought had a high degree of structural heterogeneity. This finding is in line with our previous study, in which the relevance feedback information was used to reweight the fragments. However, it should be pointed out that while using relevance feedback information is limited only by computational cost, using a set of reference structures implies the availability of bioactivities.

## Figures and Tables

**Table 1:** MDDR activity classes for DS1 dataset.

Activity index	Activity class	Active molecules	Pairwise similarity (mean)
31420	Renin inhibitors	1130	0.290
71523	HIV protease inhibitors	750	0.198
37110	Thrombin inhibitors	803	0.180
31432	Angiotensin II AT1 antagonists	943	0.229
42731	Substance P antagonists	1246	0.149
06233	Substance P antagonists	752	0.140
06245	5HT reuptake inhibitors	359	0.122
07701	D2 antagonists	395	0.138
06235	5HT1A agonists	827	0.133
78374	Protein kinase C inhibitors	453	0.120
78331	Cyclooxygenase inhibitors	636	0.108

**Table 2:** MDDR activity classes for DS2 dataset.

Activity index	Activity class	Active molecules	Pairwise similarity (mean)
07707	Adenosine (A1) agonists	207	0.229
07708	Adenosine (A2) agonists	156	0.305
31420	Renin inhibitors 1	1300	0.290
42710	CCK agonists	111	0.361
64100	Monocyclic-lactams	1346	0.336
64200	Cephalosporins	113	0.322
64220	Carbacephems	1051	0.269
64500	Carbapenems	126	0.260
64350	Tribactams	388	0.305
75755	Vitamin D analogous	455	0.386

**Table 3:** MDDR activity classes for DS3 dataset.

Activity index	Activity class	Active molecules	Pairwise similarity (mean)
09249	Muscarinic (M1) agonists	900	0.111
12455	NMDA receptor antagonists	1400	0.098
12464	Nitric oxide synthase inhibitors	505	0.102
31281	Dopamine-hydroxylase inhibitors	106	0.125
43210	Aldose reductase inhibitors	957	0.119
71522	Reverse transcriptase inhibitors	700	0.103
75721	Aromatase inhibitors	636	0.110
78331	Cyclooxygenase inhibitors	636	0.108
78348	Phospholipase A2 inhibitors	617	0.123
78351	Lipoxygenase inhibitors	2111	0.113

**Table 4:** The recall is calculated using the top 1% and top 5% of the DS1 data sets when ranked using the TAN, BIN, and BINRF.

Activity index	1%			5%		
	TAN	BIN	BINRF	TAN	BIN	BINRF
31420	55.84*	74.08*	81.8**	85.49*	87.61**	84.12*
71523	22.26*	28.26*	43.86**	42.7*	52.72*	68.72**
37110	12.54*	26.05*	41.25**	24.11*	48.2*	71.05**
31432	33.36*	39.23*	46.5**	68.2*	77.57*	91.59**
42731	16.24*	21.68*	28.13**	32.81*	26.63*	42.39**
06233	14.23*	14.06*	16.75**	27.01*	23.49*	32.93**
06245	10.06**	6.31*	10.04*	22.9*	14.86*	28.8**
07701	8.91*	11.45*	19.75**	23.1*	27.79*	41.24**
06235	11.87*	10.84*	12.45**	24.54*	23.78*	31.89**
78374	16.75*	14.25*	25.49**	24.26*	20.2*	39.18**
78331	8.05*	6.03*	8.14**	16.83**	11.8*	11.20*

Mean	**19.10**	**22.93**	**30.38**	**35.63**	**37.69**	**49.73**

Share cells	1	0	10	1	1	9

**Table 5:** The recall is calculated using the top 1% and top 5% of the DS2 data sets when ranked using the TAN, BIN, and BINRF.

Activity index	1%			5%		
	TAN	BIN	BINRF	TAN	BIN	BINRF
07707	78.3**	72.18*	72.33*	91.08**	74.81*	74.17*
07708	74.01*	96*	100**	88.52*	99.61*	100**
31420	46.44*	79.82*	82.71**	77.6*	95.46*	97.15**
42710	57.22*	76.27*	95.36**	67.59*	92.55*	99.36**
64100	93.22*	88.43**	87.75*	97.89*	99.22**	98.93*
64200	63.39*	70.18*	71.79**	89.82*	99.2**	99.12*
64220	73.56*	68.32*	82.47**	92.05*	91.32*	98.89**
64500	60.75*	81.2*	96.56**	74.98*	94.96*	99.28**
64350	76.69*	81.89*	93.67**	90.34*	91.47*	98.24**
75755	95.99*	98.06*	98.26**	98.78**	98.33*	98.33*

Mean	**71.957**	**81.235**	**88.09**	**86.86**	**93.69**	**96.34**

Share cells	1	1	8	2	2	6

**Table 6:** The recall is calculated using the top 1% and top 5% of the DS3 data sets when ranked using the TAN, BIN, and BINRF.

Activity index	1%			5%		
	TAN	BIN	BINRF	TAN	BIN	BINRF
09249	25.09**	15.33*	15.51*	40.21**	25.72*	29.08*
12455	7.7*	9.37*	11.59**	19.08**	14.65*	16.77*
12464	9.02*	8.45*	11.67**	14.56*	16.55*	27.1**
31281	27.53*	18.29*	44.48**	44*	28.29*	59.9**
43210	11.1**	7.34*	9.41*	26.37**	14.41*	21.27*
71522	2.35*	4.08*	11.39**	6.28*	8.44*	23.62**
75721	24.02*	20.41*	28.24**	28.97*	30.02*	56.39**
78331	6.27*	7.51*	10.11**	15.79*	12.03*	18.82**
78348	4.69*	9.79**	8.99*	13.16*	20.76*	24.15**
78351	4.31*	13.68*	16.64**	10.55*	12.94*	20.16**

Mean	**12.21**	**11.42**	**16.80**	**21.90**	**18.38**	**29.72**

Share cells	2	1	7	3	0	7

**Table 7:** Rankings of weighting functions based on Kendall *W* test results: DS1–DS3 Top 1% and 5%.

Dataset	Recall type	* W*	* P*	Ranking
DS1	1%	0.75	<0.01	BINRF > BIN > TAN
	5%	0.71	<0.01	BINRF > TAN > BIN

DS2	1%	0.39	>0.01	BINRF > BIN > TAN
	5%	0.28	<0.01	BINRF > BIN > TAN

DS3	1%	0.37	<0.01	BINRF>TAN>BIN
	5%	0.39	<0.01	BINRF>TAN>BIN

**Table 8:** Number of (∗ and ∗∗) cells for mean recall of actives using different search models for DS1–DS3 Top 1% and 5%.

Dataset	TAN	BIN	BINRF
Top 1%			
DS1	1	0	10
DS2	1	1	8
DS3	2	1	7

Top 5%			
DS1	1	1	9
DS2	2	2	6
DS3	3	0	7
